# Landmark analysis of the risk of recurrence after resection or ablation for HCC: A nationwide study

**DOI:** 10.1097/HC9.0000000000000472

**Published:** 2024-06-19

**Authors:** Frederik Kraglund, Nikolaj Skou, Gerda Elisabeth Villadsen, Peter Jepsen

**Affiliations:** 1 Department of Hepatology and Gastroenterology, Aarhus University Hospital, Aarhus N, Denmark; 2 Department of Radiology, Aarhus University Hospital, Aarhus N, Denmark; 3 Department of Clinical Epidemiology, Aarhus University Hospital, Aarhus N, Denmark

## Abstract

**Background:**

The risk of HCC recurrence at particular landmarks since the initial treatment is unknown. With this registry-based study, we aimed to provide a nuanced description of the prognosis following resection or ablation for HCC, including landmark analyses.

**Methods:**

Using the Danish nationwide health care registries, we identified all patients who received resection or ablation in 2000–2018 as the first HCC treatment. HCC recurrence was defined as a new HCC treatment > 90 days after the first treatment. We conducted competing risk landmark analyses of the cumulative risk of recurrence and death.

**Results:**

Among 4801 patients with HCC, we identified 426 patients who received resection and 544 who received ablation. The 2 treatment cohorts differed in cirrhosis prevalence and tumor stage. The 5-year recurrence risk was 40.7% (95% CI 35.5%−45.8%) following resection and 60.7% (95% CI: 55.9%−65.1%) following ablation. The 1-year recurrence risk decreased over the landmarks from 20.4% (95% CI: 16.6%−24.6%) at the time of resection to 4.7% (95% CI: 0.9%−13.9%) at the 5-year landmark. For ablation, the risk decreased from 36.1% (95% CI: 31.9%−40.4%) at the time of treatment to 5.3% (95% CI: 0.4%−21.4%) at the 5-year landmark. The risk of death without recurrence was stable over the landmarks following both resection and ablation.

**Conclusions:**

In conclusion, the risk of recurrence or death following resection or ablation for HCC is high from the treatment date, but the risk of recurrence decreases greatly over the survival landmarks. This information is valuable for clinicians and their patients.

## INTRODUCTION

Primary liver cancer, of which HCC comprises 75%–85%, causes over 800,000 deaths every year, making it the third most common cause of cancer-related death worldwide.^1^ Only few HCCs are amenable to curative-intent treatment such as liver resection or ablation,^2^ and even after those treatments, the overall and disease-free survival remain low.^3,4^ Clinically important aspects relating to the prognosis following resection of ablation for HCC have yet to be described: What the competing risks of recurrence and death without recurrence are and how these risks develop over time.

With this registry-based study, we aimed to provide a nuanced description of the prognosis following resection or ablation for HCC.

## METHODS

### Setting

The source population of this study was the Danish population of 5,781,190 people as of January 1, 2018. All Danish citizens are provided free, tax-supported health care with equal access to primary, secondary, and tertiary care. Through the Danish Civil Registration System, each citizen is given a unique personal identifier at birth or immigration, allowing linkage of the national registries while ensuring complete follow-up until death or emigration.^5^ The National Patient Registry records all hospital contacts including diagnoses, examinations, and procedures since 1977.^6^ The Cancer Registry records all incident cancer diagnoses since 1943, including TNM-stage since 2004.^7^ The Danish guidelines for diagnostic workup, treatment, and clinical follow-up of HCC are based on the European Association for the Study of the Liver (EASL) guidelines.^8^

### Patients

We identified all patients diagnosed with HCC from January 1, 2000 to December 31, 2018. We further identified all HCC treatments received by these patients, including liver resections; radiofrequency, microwave, and chemical ablation treatments for HCC; liver transplantations; transarterial chemoembolization; selective internal radiation therapies; stereotactical body radiation therapies; systemic treatments; and palliative treatments. All codes used to define HCC treatments are listed in Supplemental Table S1, http://links.lww.com/HC9/A939.

To assess whether the study population defined from January 1, 2000 to December 31, 2018 represented the current patient population, we repeated our analyses using a restricted population of patients diagnosed with HCC from January 1, 2014 to December 31, 2018.

Patients whose first HCC treatment was resection were included in the resection cohort on the date of the treatment, and patients whose first HCC treatment was ablation were included in the ablation cohort on the date of the treatment. If a patient received a combination of resection and intraoperative ablation, the surgery was classified as a resection. If a patient received both ablation and transarterial chemoembolization treatments within 3 days, the first treatment was considered a combination therapy of ablation and transarterial chemoembolization, and the patient was excluded. The study was approved by the National Board of Health and by the Danish Data Protection Agency (Journal No. 1-16-02-321-19). According to Danish law, approval from the Danish Committee on Health Research Ethics was not necessary, and written consent was not required.

### Cirrhosis

According to Danish and international guidelines, an HCC diagnosis can only be made without biopsy verification if the patient has cirrhosis. Therefore, patients with HCC who have not had a liver biopsy must have cirrhosis. Hence, cirrhosis was defined as either having a diagnostic code for liver cirrhosis or not having had a liver biopsy performed before or on the date of the first resection or ablation (Supplemental Table S1, http://links.lww.com/HC9/A939).

### Outcomes

HCC recurrence was defined as receiving any HCC treatment or being referred to oncology or palliative medicine more than 90 days after the initial resection or ablation. We chose this 90-day threshold because it is the recommended time interval between HCC resection and the first recurrence assessment by CT scan according to both international^8^ and Danish guidelines. For ablation, Danish guidelines recommend CT scan or MR scan 4–6 weeks after the first ablation. We also used the 90-day threshold for ablation for the purpose of obtaining comparable analyses, and because any HCC treatment within 90 days of the initial treatment is likely due to technical failure of the initial treatment rather than a recurrence treatment. However, the clinical difference between treatment failure and HCC recurrence is indistinct. Therefore, to investigate whether the choice of threshold would influence our results and conclusions, we conducted sensitivity analyses on the recurrence risk using the alternative thresholds: 30, 60, and 120 days. Dates of death were obtained from the Central Office of Civil Registration.

### Statistics

All-cause mortality was computed using the Kaplan-Meier estimator. In a competing risk setting, the cumulative incidence (ie, the cumulative risk) of recurrence was computed regarding death without recurrence as a competing event, and vice versa. Similarly, the cumulative risk of dying from a given cause was computed with death from any other cause as a competing event. We utilized competing risk analyses because the estimates reflect the actual risk of recurrence and of death without recurrence. Any noncompeting risk analysis would overestimate these risks.^9^ We used Stata 17 (StataCorp. 2021. Stata Statistical Software: Release 17. College Station, TX: StataCorp LLC.) and the packages “stcompet,” “stcomlist,” “stcrreg,” and “stcrprep” to conduct the competing risks analyses. Landmark analyses (N = 60) were conducted, treating each month since the first HCC treatment as a landmark.^10^ Thus, for each month of follow-up, the 1- and 5-year risks of recurrence and of death without recurrence were computed in patients who were still alive and recurrence-free at that time. Landmark analyses, or “conditional prognosis” analyses, are analyses of the prognosis for patients who have remained alive and recurrence-free for some time. Thus, it describes the prognosis conditional on being alive and recurrence-free up to a specific time point. For example, a 1-year landmark analysis includes those patients who remain alive and recurrence-free one year after resection, and then it describes the prognosis for those patients onwards.

To evaluate the robustness of our landmark analysis results, we additionally conducted sensitivity analyses using the smoothed hazard function for resection and ablation. We graphed the hazard of recurrence and of death without recurrence over time since the curative-intent treatment with the purpose of comparing these functions to the landmark analysis graphs.

## RESULTS

**FIGURE 1:**
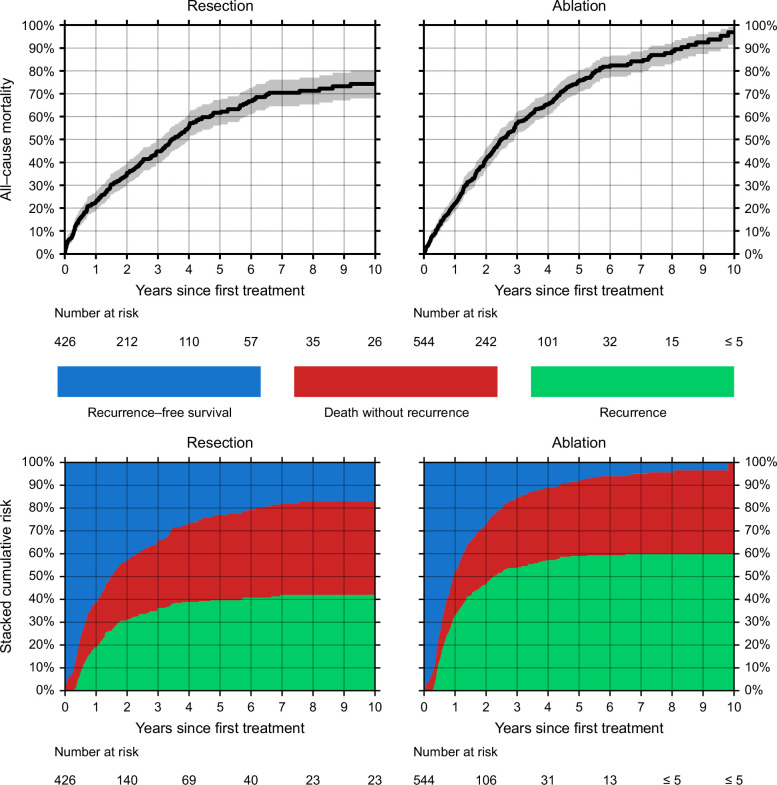
All-cause mortality (top) and stacked cumulative incidence of recurrence and death without recurrence (bottom) 10 years following resection (left) or ablation (right) for HCC.

We identified 4801 patients diagnosed with HCC in 2000–2018. Of these, 426 (8.9%) underwent resection as first-line HCC treatment and 544 (11.3%) underwent ablation. The resection and ablation cohorts were comparable according to sex, age group, and calendar period, but there were fewer patients with cirrhosis in the resection cohort (22.8% vs. 71.3%), and of the patients with known TNM-stage, those in the resection cohort had larger tumors (T stage [TNM] T3 or T4: 39.8% vs. 24.4%) (Table 1). All-cause mortality was similar in the 2 groups 1 year after resection/ablation (resection: 22.7%, 95% CI: 18.9%−27.1%; ablation: 22.0%, 95% CI: 18.6%−25.8%), but the 5-year mortality was lower in patients treated with resection (resection: 61.7%, 95% CI: 56.1%−67.3%; ablation: 75.5%, 95% CI: 70.6%−80.0%), and so was the 10-year mortality (resection: 74.3%, 95% CI: 67.9%−80.3%; ablation: 96.9%, 95% CI: 91.4%−99.2%) (Figure 1, top).

**TABLE 1:** Baseline characteristics of the study population

	Resection	Ablation
	N = 426	N = 544
Male gender, N (%)	308 (72.3)	428 (78.7)
Age category, N (%)		
< 60	118 (27.7)	145 (26.7)
60–69	148 (34.7)	218 (40.1)
70–79	132 (31.0)	140 (25.7)
≥ 80	28 (6.6)	41 (7.5)
T stage (TNM), N (% of known)		
T1	82 (26.1)	126 (37.4)
T2	107 (34.1)	129 (38.3)
T3	101 (32.2)	70 (20.8)
T4	24 (7.6)	12 (3.6)
Unknown T stage (Tx), N (%)	112 (26.3)	207 (38.1)
Cirrhosis, N (%)	97 (22.7)	388 (71.3)
Calendar period, N (%)		
2000–2004	60 (14.1)	51 (9.4)
2005–2009	60 (14.1)	84 (15.4)
2010–2014	143 (33.6)	174 (32.0)
2015–2018	163 (38.3	235 (43.2)

### Recurrence risk

After resection, the 5-year risk of recurrence was 40.7% (95% CI: 35.5%−45.8%), and the 5-year risk of death without recurrence was 36.2% (95% CI: 31.1%−41.4%), leaving 23.1% of patients alive and HCC-free after 5 years (Figure 1, bottom left). The risk of recurrence was higher after ablation: The 5-year risk was 60.7% (95% CI: 55.9%−65.1%), while the 5-year risk of death without recurrence was 31.4% (95% CI: 27.1%−35.7%), leaving 7.9% alive and HCC-free (Figure 1, bottom right). After 10 years, the probability of being alive and HCC-free was 17.3% (95% CI: 12.8%−23.3%) following resection and 0% (95% CI: 0%−6.5%) following ablation (Figure 1, bottom). The estimates of recurrence risk were only marginally influenced by the recurrence definition threshold as evidenced by our sensitivity analyses (Supplemental Table S2, http://links.lww.com/HC9/A939).

### Landmark analyses

**FIGURE 2:**
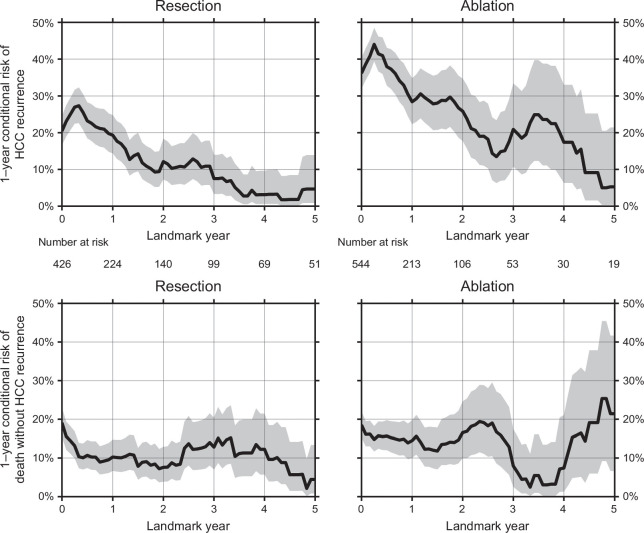
Landmark analyses of the 1-year conditional risk of recurrence (top) and death without recurrence (bottom) following resection (left) or ablation (right) for HCC. The grey areas display 95% CIs. For example, a patient who remains alive and recurrence-free 2 years after resection has a 1-year risk of recurrence of around 10%. At landmark year 5, this risk has further decreased to around 5% (top left, landmark years 2 and 5).

We found that the 1-year risk of recurrence at the 1-year landmark was 19.3% (95% CI: 14.4%−24.8%), meaning that the risk of recurrence within the next year is essentially the same for patients who have just been resected as for patients who remain HCC-free 1 year later (Figure 2, top left, time points 0 and 1 year). After that, the risk decreased. Specifically, the 1-year risk of recurrence decreased from 20.4% (95% CI: 16.6%−24.6%) at the time of resection to 4.7% (95% CI: 0.9%−13.9%) at the 5-year landmark, meaning that patients who have remained recurrence-free for 5 years after resection have a 1-year risk of recurrence that is one-quarter of what it was right after resection (Figure 2, top left). After ablation, the 1-year recurrence risk decreased from 36.1% (95% CI: 31.9%−40.4%) at the time of ablation to 5.3% (95% CI: 0.4%−21.4%) at the 5-year landmark (Figure 2, top right). The 1-year risk of death without recurrence decreased from 19.1% (95% CI: 15.4%−23.0%) at the time of resection to 4.5% (95% CI: 0.8%−13.4%) at the 5-year landmark, and it increased from 18.5% (95% CI: 15.3%−22.0%) at the time of ablation to 21.4% (95% CI: 6.6%−41.7%) at the 5-year landmark (Figure 2, bottom).

The chance of recurrence-free survival can be computed by subtracting from 100% the risk of recurrence and the risk of death without recurrence. Thus, the 1-year recurrence-free survival increased from 60.5% (95% CI: 52.4%−68.0%) at the time of resection to 91.0% (95% CI: 72.7%−98.3%) at the 5-year landmark, meaning that patients who were alive and recurrence-free 5 years after resection had a 91% chance of still being alive and recurrence-free 1 year later. The 1-year recurrence-free survival increased from 45.3% (95% CI: 37.6%−52.8%) at the time of ablation to 73.3% (95% CI: 36.9%−93.0%) at the 5-year landmark.

### Sensitivity analyses

The smoothed hazard functions showed similar patterns of the risks of recurrence and death without recurrence over time since the initial treatment (Supplemental Figure S1, http://links.lww.com/HC9/A939). For both the landmark analysis graphs and the smoothed hazard functions, there was a high risk (or hazard) of recurrence within the first year. For resection, there was a slight peak in risk after 3 years. For ablation, there were 2 peaks in recurrence risk at 2 and 4 years (Figure 2, top and Supplemental Figure S1, http://links.lww.com/HC9/A939). Using the restricted 2014–2018 study population, the results of the recurrence risk and landmark analyses were similar, although the same number of follow-up years was unachievable (Supplemental Figure S2, http://links.lww.com/HC9/A939, and S3, http://links.lww.com/HC9/A939).

## DISCUSSION

With this nationwide study, we confirmed that the risk of HCC recurrence and of death without HCC is high in patients treated with resection or ablation for HCC. Five years after resection, only 23.1% of patients were alive and recurrence-free, and the corresponding proportion after ablation was just 7.9%. Fortunately, the chance of recurrence-free survival increased with each posttreatment landmark reached, so that patients who were still alive and without recurrence 5 years after their initial resection or ablation had a much better prognosis: From that 5-year landmark, resected patients had a 4.7% 1-year risk of recurrence and a 4.5% 1-year risk of death without recurrence, while ablated patients had a 5.3% 1-year risk of recurrence and a 21.4% 1-year risk of death without recurrence. Importantly, the resection and ablation cohorts were very different regarding cirrhosis prevalence and tumor size. Therefore, the results of this study should not be interpreted as a comparison between resection and ablation.

Our estimates of all-cause mortality from the time of resection/ablation are in line with the existing literature.^11^ However, comparisons of prognosis measures across studies are complicated by vast differences between study populations: the prevalence of portal hypertension, underlying liver disease, poor liver function, vascular invasion, and progressed tumor stages.^12^ Geographical variations in the prevalence of liver diseases, HCC staging, and treatment further complicate any attempts of comparing estimates.^2^ The risk of recurrence observed in our study was lower compared to prior reports.^13–15^ However, prior studies have not regarded death without recurrence as a competing event and have, therefore, overestimated the risk of recurrence.^9^ Consequently, this is the first study to estimate the actual or “real-world” risk of recurrence following resection or ablation for HCC.

Using landmark analyses, we found that the risk of recurrence decreased over the landmarks after both resection and ablation, while the risk of death without recurrence was stable over the landmarks. This finding was replicated using smoothed hazard functions. Reviewing the literature, we found no prior landmark analyses of the risk of recurrence or of death without recurrence following curative-intent treatments for HCC. However, 5 prior studies have conducted survival landmark analyses following resection,^16–20^ and one of these studies also presented landmark analyses following ablation.^20^ The patient populations in the 5 studies differ substantially in geography, HCC stage, HCC grade, cirrhosis, tumor size, and microvascular invasion, complicating attempts to compare their results. With that caveat in mind, the existing literature generally indicates a stable survival over the landmarks following both resection and ablation, and that is consistent with what we show in Figure 2 (bottom) and Supplemental Figure S1, http://links.lww.com/HC9/A939.

The first potential limitation of this registry-based study is the lack of clinical information on liver function (eg, Child-Pugh score), performance status, and, in some patients, tumor stage. The second is the validity of codes in the registries. Every patient included in the treatment cohorts was defined using at least 2 registry codes: 1 code for HCC and 1 code for liver resection or ablation. The Danish Cancer Registry has a very high validity^21^ owing to mandatory reporting of incident cancer cases and continual validation of the registry.^7^ Furthermore, the codes for cancer in the National Patient Registry have an overall high positive predictive value of 98.0% (89.4%–99.9%).^22^ The codes for resection and ablation have not been specifically validated, but the PPV of codes for other gastrointestinal surgeries codes range between 95.9% (93.2%–97.5%)^23^ for i.p. adhesiolysis and 99.9% (99.6%–100%) for cholecystectomy.^24^ Surgical procedures are coded immediately after the procedure, which might explain the high validity of these codes. Lastly, the coding practices in Denmark have not formally been altered throughout the study period of 2000–2018.^6^ Altogether, it is highly unlikely that our results are biased by misclassification of patients. The third limitation is the potential heterogeneity of treatments and surgical techniques over the study period of 2000–2018. For instance, the introduction of direct-acting antivirals in 2014 significantly improved the prognosis of liver disease caused by hepatitis C. However, our findings were robust to restricting the study period to 2014–2018. The fourth limitation is the potential misclassification of HCC recurrence. We defined HCC recurrence as any HCC treatment (excluding liver transplantation) that occurred > 90 days after the initial treatment. Therefore, any HCC treatments performed within this 90-day threshold were not counted as recurrences. The use of a threshold (in our case, a 90-day threshold) inevitably introduces potential misclassification of true recurrences as treatment failures and vice versa. However, our analyses were robust to changes in the defined recurrence threshold. Another limitation of using HCC treatments as a proxy for recurrence is that some recurrences might not be identified if a patient died of hepatic decompensation before receiving any treatment. However, we also included referral to oncology departments and palliative care in our definition of recurrence, reducing the potential for misclassification of true recurrences as deaths without recurrence.

This study highlights that the prognosis of patients treated with resection or ablation for HCC is not fixed from the date of the treatment: The risk of recurrence is initially high after resection or ablation for HCC but decreases substantially over each landmark since the treatment. This insight is useful for patients and their hepatologists. Additionally, it is useful at a treatment center level to optimize clinical follow-up of patients with HCC, and to estimate the cost-effectiveness of imaging intervals past different landmarks. For instance, the risk of recurrence is very low after the 3-year landmark following resection, indicating that the control visits following that landmark could be scheduled less frequently. Alternatively, different imaging modalities could be utilized according to the a priori risk of recurrence. After the first critical year of CT/MRI-based follow-up, it could be considered to return to an ultrasound-based surveillance program. The fact that the risk of death without HCC was stable over the landmarks underlines that HCC treatments can only be expected to treat HCC and not any underlying liver disease. Therefore, patients with HCC and underlying cirrhosis should be clinically followed and treated for both diseases.

In conclusion, the all-cause mortality and risk of recurrence were high after curative-intent resection or ablation for HCC. However, the risk of recurrence decreased over the landmarks while the risk of dying without recurrence remained stable. This information is valuable to clinicians and their patients, and it should be helpful in guiding clinical follow-up following curative-intent HCC treatment.

## Supplementary Material

SUPPLEMENTARY MATERIAL
