# Supplementary material for: Landmark analysis of the risk of recurrence after resection or ablation for HCC: A nationwide study
Source: Hepatol Commun. 2024 Jun 19;8(7):e0472. doi: 10.1097/HC9.0000000000000472 (PMC11186808; doi:10.1097/HC9.0000000000000472)
Supplement: SUPPLEMENTARY MATERIAL [file hc9-8-e0472-s001.docx]

# Supplementary material

Landmark analysis of the risk of recurrence after resection or ablation for hepatocellular carcinoma: a nationwide study

Frederik Kraglund, Nikolaj Skou, Gerda Elisabeth Villadsen, Peter Jepsen

## Table of contents

Supplementary Table S1…………………………………………………………………………….. 2

Supplementary Table S2…………………………………………………………………………….. 4

Supplementary Figure S1……………………………………………………………………………. 5

Supplementary Figure S2……………………………………………………………………………. 6

Supplementary Figure S3……………………………………………………………………………. 7

**Supplementary Table S1.** Registry codes used to identify the study population, HCC treatments, aetiologies, and cirrhosis.

| **Danish National Patient Registry** |  |  |  |
| --- | --- | --- | --- |
|  | **Diagnoses** |  |  |
|  |  | Hepatocellular carcinoma | ICD-10: C22.0x |
|  |  | Liver cirrhosis | ICD-10: K70.3x, K71.7x, K74.3x-K74.6x |
|  |  | Ascites | ICD-10: R18x |
|  |  | Spontaneous bacterial peritonitis | ICD-10: K65.8I |
|  |  | Hepatorenal syndrome | ICD-10: K76.7 |
|  |  | Variceal bleeding | ICD-10: I85.0, I86.4A |
|  |  | Diabetes and complications of diabetes | ICD-10: E10x-E14x, G63.2, H36.0x, N08.3 |
|  | **Surgical procedures** |  |  |
|  |  | Liver transplantation | NCSP: JJC 00, JJC 10, JJC 20, JJC 30, JJC 40 |
|  |  | Liver resection | NCSP: JJB xx, JJA 40, JJA 41 |
|  |  | Ablation of pathological liver tissue | NCSP: JJA 43x, JJA 44, TJJ 10 |
|  |  | Transarterial chemoembolization (TACE) or selective internal radiation therapy (SIRT) | NCSP: PCT 20; DCT: WGG2A, WHA110, WHA102 |
|  |  | Treatment of oesophageal varices | NCSP: JCA 20, JCA 22, JCA 32 |
|  |  | Ascites drainage | NCSP: TJA 10x |
|  | **Non-surgical treatments** |  |  |
|  |  | Treatment with Sorafenib | DCT: WHA407 |
|  |  | External beam radiation therapy | DCT: WGC22 |
|  |  | Best supportive care | DCT: XB xx |
|  | **Referrals** |  |  |
|  |  | Referral to oncology or palliative medicine | Hospital department-classification: 022, 014 |
| **Danish Cancer Register** |  |  |  |
|  | **Diagnoses** |  |  |
|  |  | Hepatocellular carcinoma | ICD-10: C22.0x |
|  | **TNM codes** |  |  |
|  |  | TNM-T | ICD-10: AZCD13x-AZCD16x, AZCD19, AZCE13x-AZCE16x, AZCE19 |
|  |  | TNM-N | ICD-10: AZCD30x-AZCD33x, AZCD39, AZCE30x-AZCE33, AZCE39 |
|  |  | TNM-M | ICD-10: AZCD40x-AZCD41x, AZCD49, AZCE40x-AZCE41x, AZCE49 |

**Supplementary Table S2.** Results from the sensitivity analyses using different recurrence definition cut-offs (30 days, 60 days, 90 days, 120 days).

|  |  | Recurrence definition cut-off | | | |
| --- | --- | --- | --- | --- | --- |
|  |  | 30 days | 60 days | 90 days (primary analysis) | 120 days |
| Resection | |  |  |  |  |
|  | 1-year recurrence risk (95% CI) | 21.9% (17.9−26.0%) | 21.2% (17.3−25.3%) | **20.5% (16.6−24.6%)** | 18.8% (15.1−22.8%) |
|  | 5-year recurrence risk (95% CI) | 41.6% (36.4−46.8%) | 41.2% (36.0−46.3%) | **40.7% (35.5−45.8%)** | 39.6% (34.5−44.7%) |
| Ablation | |  |  |  |  |
|  | 1-year recurrence risk (95% CI) | 40.1% (35.8−44.4%) | 38.7% (34.4−42.9%) | **36.1% (31.9−40.4%)** | 32.9% (28.6−37.3%) |
|  | 5-year recurrence risk (95% CI) | 63.5% (58.7−67.8%) | 62.5% (58.0−66.9%) | **60.7% (55.9−65.1%)** | 59.0% (54.2−63.4%) |

**Supplementary Figure S1.** Smoothed functions of the hazards of HCC recurrence (top) and death without recurrence (bottom) following resection (left) and ablation (right).


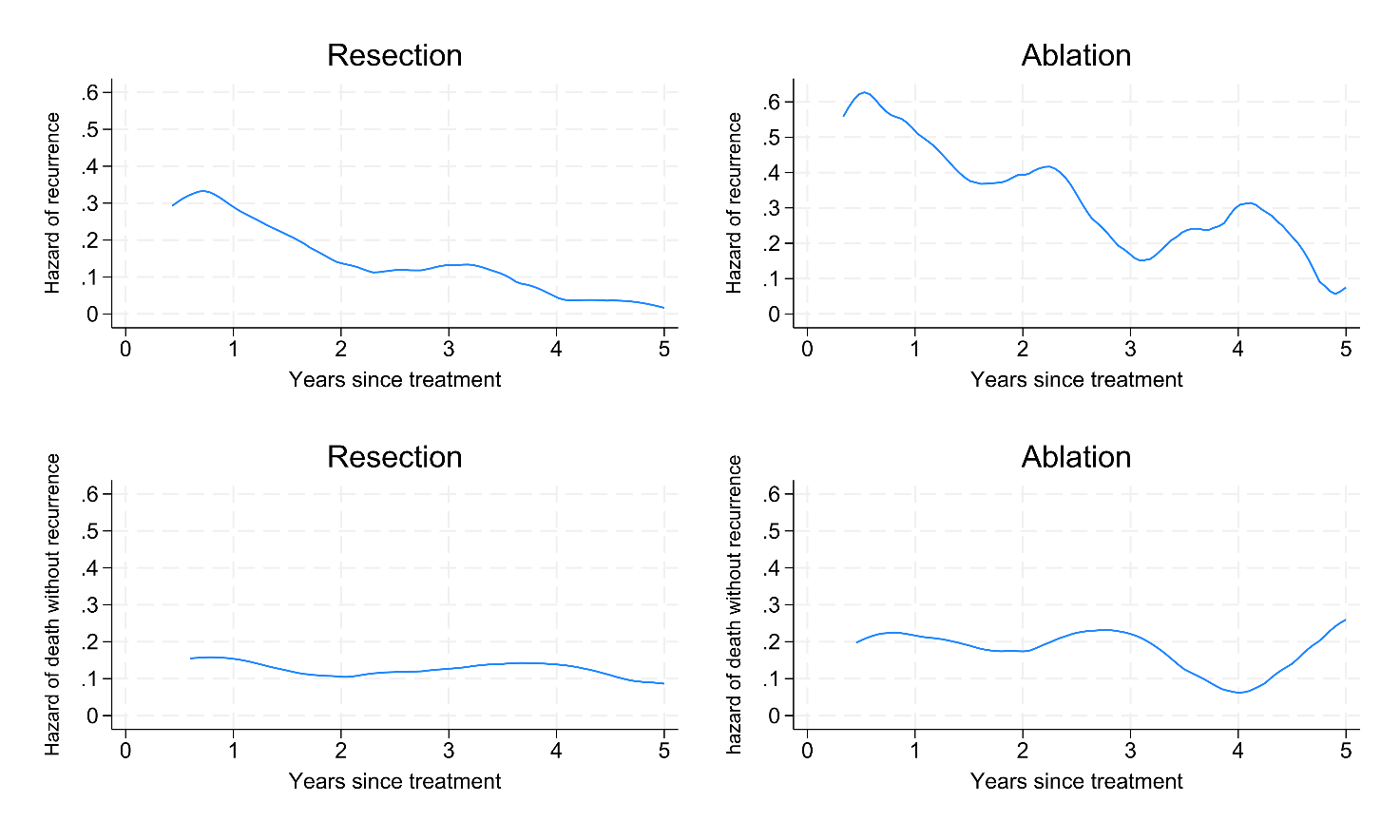


**Supplementary Figure S2**. Sensitivity analysis restricted to 2014-2018. All-cause mortality (top) and stacked cumulative incidence of recurrence and death without recurrence (bottom) 10 years following resection (left) or ablation (right) for HCC.


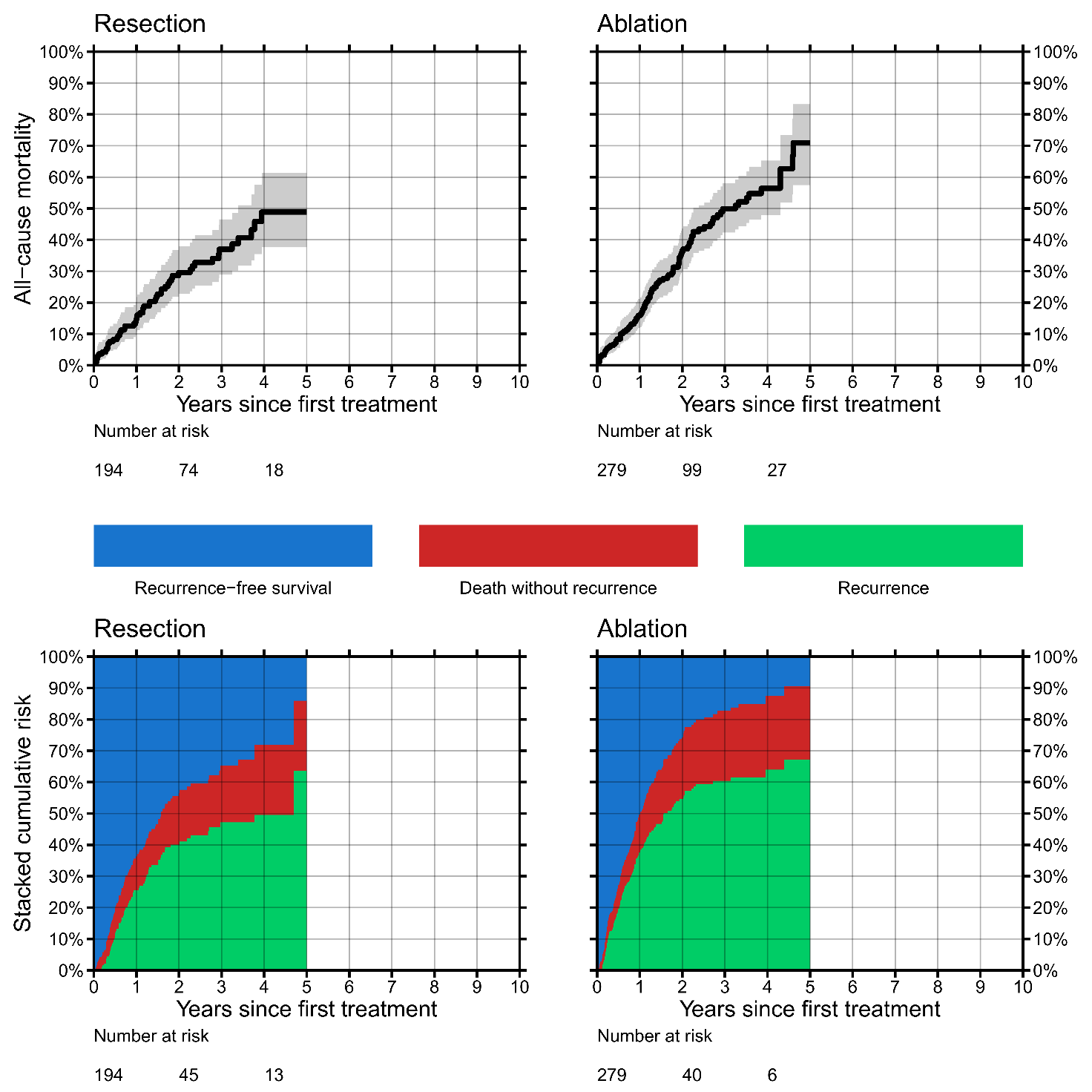


**Supplementary Figure S3.** Sensitivity analysis restricted to 2014-2018. Landmark analyses of the 1-year conditional risk of recurrence (top) and death without recurrence (bottom) following resection (left) or ablation (right) for HCC. The grey areas display the 95% confidence intervals.

**
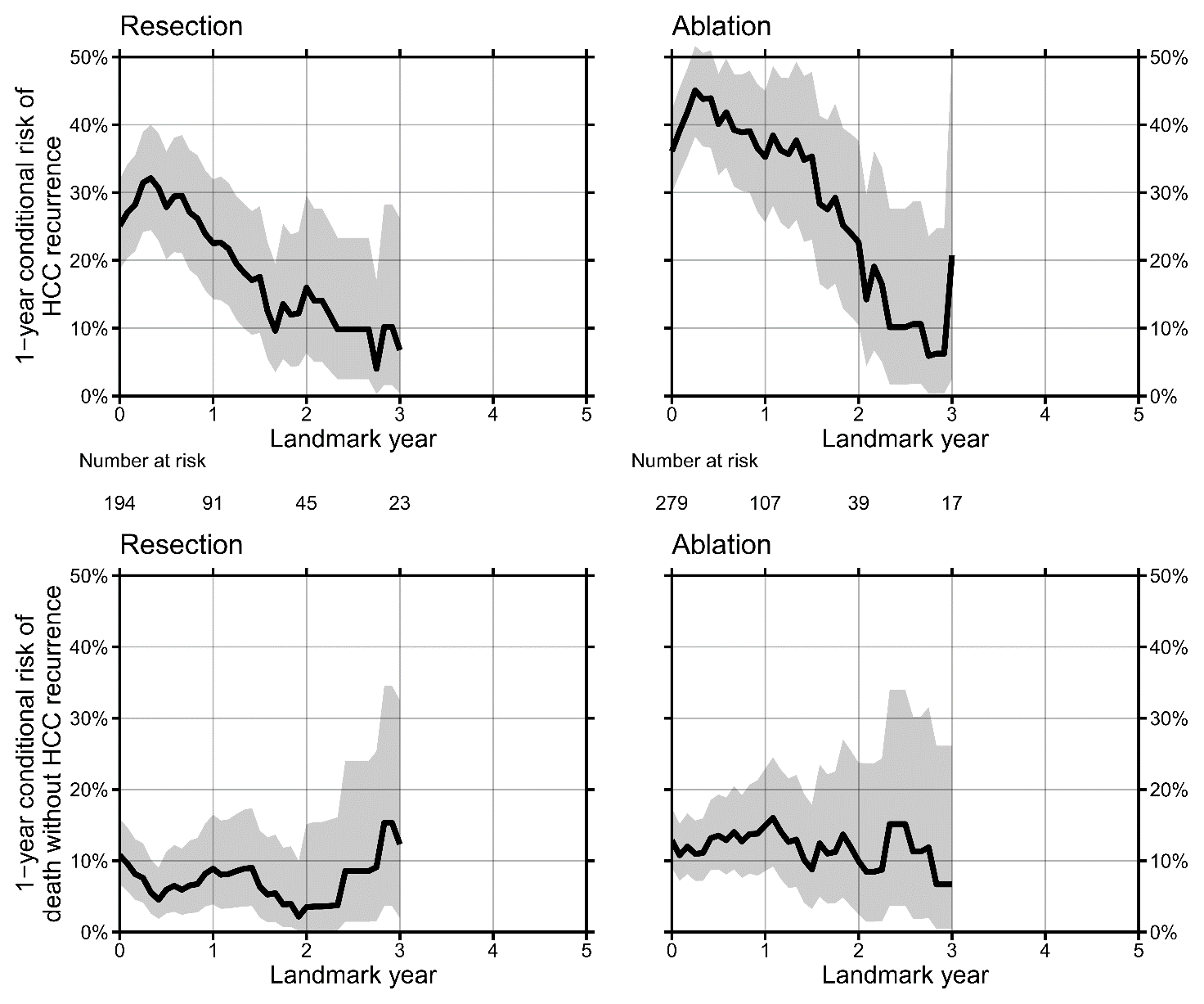
**
